# Phylogeographic Genetic Diversity in the White Sucker Hepatitis B Virus across the Great Lakes Region and Alberta, Canada

**DOI:** 10.3390/v13020285

**Published:** 2021-02-12

**Authors:** Cynthia R. Adams, Vicki S. Blazer, Jim Sherry, Robert Scott Cornman, Luke R. Iwanowicz

**Affiliations:** 1 Fish Health Branch, Leetown Science Center, US Geological Survey Kearneysville, WV 25430, USA; cindy.adams@pitt.edu (C.R.A.); vblazer@usgs.gov (V.S.B.); 2 Environment and Climate Change Canada, Burlington, ON 7S 1A1, Canada; jim.sherry@ca.gov; 3 Fort Collins Science Center, US Geological Survey, Fort Collins, CO 80526, USA; rcornman@usgs.gov

**Keywords:** hepadnaviruses, hepatitis B virus, white sucker, Great Lakes, phylogeography, haplotypes, fish diseases

## Abstract

Hepatitis B viruses belong to a family of circular, double-stranded DNA viruses that infect a range of organisms, with host responses that vary from mild infection to chronic infection and cancer. The white sucker hepatitis B virus (WSHBV) was first described in the white sucker (*Catostomus commersonii*), a freshwater teleost, and belongs to the genus *Parahepadnavirus*. At present, the host range of WSHBV and its impact on fish health are unknown, and neither genetic diversity nor association with fish health have been studied in any parahepadnavirus. Given the relevance of genomic diversity to disease outcome for the orthohepadnaviruses, we sought to characterize genomic variation in WSHBV and determine how it is structured among watersheds. We identified WSHBV-positive white sucker inhabiting tributaries of Lake Michigan, Lake Superior, Lake Erie (USA), and Lake Athabasca (Canada). Copy number in plasma and in liver tissue was estimated via qPCR. Templates from 27 virus-positive fish were amplified and sequenced using a primer-specific, circular long-range amplification method coupled with amplicon sequencing on the Illumina MiSeq. Phylogenetic analysis of the WSHBV genome identified phylogeographical clustering reminiscent of that observed with human hepatitis B virus genotypes. Notably, most non-synonymous substitutions were found to cluster in the pre-S/spacer overlap region, which is relevant for both viral entry and replication. The observed predominance of p1/s3 mutations in this region is indicative of adaptive change in the polymerase open reading frame (ORF), while, at the same time, the surface ORF is under purifying selection. Although the levels of variation we observed do not meet the criteria used to define sub/genotypes of human and avian hepadnaviruses, we identified geographically associated genome variation in the pre-S and spacer domain sufficient to define five WSHBV haplotypes. This study of WSHBV genetic diversity should facilitate the development of molecular markers for future identification of genotypes and provide evidence in future investigations of possible differential disease outcomes.

## 1. Introduction

Hepadnaviruses are enveloped, partially double-stranded, hepatotrophic DNA viruses. Until recently, this family was defined by features of the two extant genera (ortho- and avihepadnavirus, which infect mammals and birds, respectively). The discovery of non-mammalian, non-avian hepadnaviruses has revealed diverse genomes with a complex evolutionary history [1,2,3]. While these newly recognized hepadnaviruses of fish share conserved genomic organization of the core, polymerase and surface open reading frames (ORFs), novel genomic features are evident as well. For instance, their genomes are larger (~10%) than ortho- or avihepadnaviruses (~3200 bp), and this additional genomic material contains putative ORFs for hypothetical proteins or is non-protein-coding. This contrasts with the prototypical hepadnaviral genome, which is completely protein-coding; however, putative small ORFs not observed in ortho- and avihepadnaviral genomes have also been identified in more recently described hepadnaviruses [1]. 

The pathogenicity of hepadnaviruses has primarily been studied in humans. Orthohepadnaviruses are associated with human liver diseases such as cirrhosis and hepatocellular carcinoma, while avihepadnaviruses typically cause transient or chronic liver infections with minimal pathogenicity [4]. Human hepatitis B (hHBV) infection is recognized as the leading risk factor for the development of liver cancer globally, and, as of 2017, ~2.9% of the human population was infected with hHBV [5]. Hepadnaviral virulence is affected by host-specific factors, environmental factors, and viral biology. These factors include the host age of exposure, route of infection, immune competence, and environmental factors [6]. Viral quasispecies also influence the effectiveness of viral clearance and host recovery [7]. In the case of human hepadnaviruses, there are 10 defined genotypes that are differentially associated with liver disease. These 10 genotypes are phylogeographically clustered [8,9] and each genotype is characterized by differences in prevalence, transmission, replication rate, serological profile, and clinical outcome [10,11,12]. The hHBV genotypes are characterized by whole-genome sequence divergence of >8% and subgenotypes differ from each other by 4–8% [8].

The disease outcomes of hepadnavirus infection in fishes and herptile hosts is unknown, and virulence factors or disease associations have not been studied, given the recent discovery of these viruses. The recently described white sucker hepadnavirus (WSHBV) provides an opportunity for probing potential disease associations between a fish hepadnavirus and its host, including genetic and environmental correlates.

White suckers are sentinel species in the Great Lakes region (USA and Canada) that present tumors and other deformities relatively frequently; these are interpreted as indicators of degraded environments associated with exposure to chemical contaminants [13,14]. Prevalence of liver tumors is a biological metric (beneficial use impairment) used to assess Areas of Concern. While there has been insufficient research to establish whether WSHBV infection is a liver tumor risk factor, a prerequisite of such research is a working understanding of WSHBV genomic diversity. This requirement relates to both the practical need for accurate assessment of WSHBV by polymerase chain reaction and the empirical demonstration that haplotype variation modulates a pathogenicity similar to that of hHBV. Here, we establish a methodological workflow to identify virus-positive fish and sequence WSHBV genomes. In addition, we identify geographic and genetic patterns of genome diversity. The molecular methods developed and applied here can be used to determine viral prevalence and facilitate a better understanding of WSHBV and white sucker disease ecology. 

## 2. Materials and Methods

### 2.1. Sampling and Extractions

**Figure 1:**
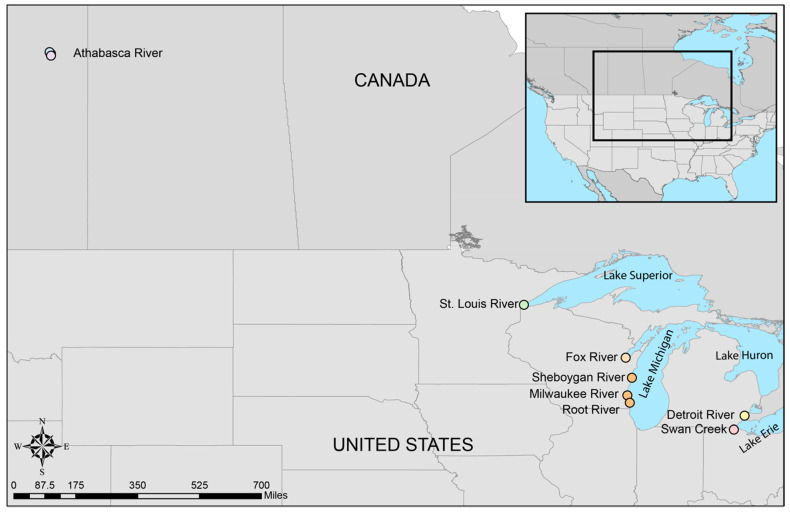
Sampling locations of white sucker within the Great Lakes and Lake Athabasca Basins. Color coding of sampling sites is retained in subsequent figures.

White sucker (*n* = 208) were collected from seven tributaries within the Great Lakes Basin between 2010 and 2012, including Areas of Concern (Figure 1). Great Lake drainages represented by these fish included that of Lake Michigan, Lake Superior and Lake Erie. Additionally, 11 white sucker were collected from two locations on the Athabasca River in Alberta Canada. Fish collection methods and IACUC are described elsewhere [13,14,15]. All fish were adults and greater than 350mm in length. Liver samples were preserved in RNAlater™. Liver or plasma were collected from these fish and stored at −80 °C until extraction of nucleic acids. Both liver and plasma were only collected from fish inhabiting the Sheboygan River. Plasma was extracted using the DNeasy Blood and Tissue Kit (Qiagen, Valencia, CA, USA), following the manufacturer instructions for nucleated blood. DNA from liver tissues preserved in ethanol was extracted using the DNeasy Blood and Tissue Kit (Qiagen, Valencia, CA, USA), following manufacturer instructions. DNA from RNAlater™ preserved liver tissue was also extracted using the DNeasy Blood and Tissue Kit, but following the Qiagen user developed protocol for purification of total DNA from soft tissues (http://www.qiagen.com/us/resources/download.aspx?id=7684840d-96bd-47a6-9d84-80cae16bf0e7&lang=EN&ver=3, accessed on 11 January 2021). 

### 2.2. Quantitative PCR

Extracted DNA from plasma and liver tissue was screened for the presence of WSHBV using quantitative PCR (qPCR). Primers for qPCR were designed using Primer3 in Geneious (v. 9.0.1, Biomatters, Auckland, New Zealand) to amplify a 115 bp fragment of the RT domain of the P ORF and S domain of S ORF (nt 1430-1544; NC_027922). The primers ccHepB_4F (5′ GACCTTAGTGAGGCGTTCTAT 3′) and ccHepB_4R (5′ CAACTCCCATAGCCGCTTTA 3′) were used at a concentration of 0.3 µM in a reaction mixture of 1X SYBR Green PCR Master Mix (Applied Biosystems, Foster City, CA, USA), 2 µL of DNA. Standards were made using PCR amplicons of the reference sample RR173 (Accession # NC_027922) and run as 10-fold serial dilutions ranging from 10^8^ copies to 1 copy. Reactions were run on an ABI Viia7 (Applied Biosystems, Foster City, CA, USA) using the conditions of 2 min at 50 °C, 10 min at 95 °C, followed by 40 cycles of 15 s at 95 °C, 1 min at 60 °C. Melt curve analysis was also performed to confirm a single product with conditions of 15 s at 95 °C and cooling at a rate of 1.6 °C/s, followed by 1 min at 60 °C and 15 s at 95 °C.

### 2.3. Long Range PCR and Next Generation Sequencing

A total of 35 virus-positive plasma (*n* = 19) and liver (*n* = 16) samples were subjected to template specific, long-range PCR (lrPCR) with primers 1265R (5′ CACCACCAGTAACACGACGA 3′) and 1488F (5′ TGGTATCTGATGGCCTGGGA 3′). Primers were designed using Primer3 in Geneious (v. 9.0.1, Biomatters, Auckland, New Zealand) to amplify an approximately 3319 bp product of the published WSHBV genome (National Center for Biotechnology Information (NCBI) accession NC_027922; nt 1488–1265). The Takara PrimeSTAR GXL DNA Polymerase (Takara Bio, Otsu, Shiga, Japan) kit was used for long-range amplification. Reactions contained 1X PrimeSTAR GXL Buffer, 250 μM dNTP Mixture, 0.15 µM forward and reverse primers, 1.25 U of PrimeSTAR GXL DNA Polymerase, 2.5 µL of template DNA and water to 25 µL. The cycling profile consisted of 30 cycles of 10 s at 98 °C, 10 s at 57 °C, and 5 min at 68 °C. In order to increase the quantity of amplified product, 1 uL of product from the lrPCR reaction was used as template for an additional 25 µL reaction containing the same components as before. This lrPCR reaction was carried out for 30 cycles of 10 s at 98 °C, 10 s at 60 °C, and 5 min at 68 °C. The lrPCR amplicon products were confirmed using gel electrophoresis on 1% I.D.NA agarose (FMC Bioproducts, Rockland, ME, USA) and stained with GelRed (Phenix, Candler, NC, USA). A subset of these products were evaluated for amplification quality on an Agilent Bioanalyzer (Agilent, Santa Clara, CA, USA). 

Amplification products from the lrPCR reactions utilizing template from plasma samples (*n* = 19) or ethanol-preserved livers (*n* = 2) were quantified using a Qubit 2.0 Fluorometer with the Qubit dsDNA HS Assay Kit (Invitrogen, Carlsbad, CA, USA). The lrPCR product was normalized to 0.2 ng/µL using 10 mM Tris-HCl, pH 8.5. Liver samples collected from fish inhabiting the Sheboygan River were handled separately. Visually verified lrPCR products from liver samples preserved in RNAlater™ (Ambion, Carlsbad, CA, USA) from the Sheboygan River (14 of 20 samples) were cleaned prior to quantification using a 3X Kapa Pure Beads (Kapa Biosystems, Wilmington, MA, USA) clean up. Long-range PCR amplicons were quantified as described above. Average size distribution of Sheboygan River samples was determined using the Agilent BioAnalyzer (Agilent, Santa Clara, CA, USA). For these Sheboygan River samples, amplicons spanning the long-range primer sites as well as for the atypical region were amplified separately for each sample using the primer sets 1019F and 1520R or 2526F and 3295R, respectively [2]. Long-range PCR amplicons and the additional two sets of amplicons were normalized in 10 mM Tris-HCl, pH 8.5 and pooled at a 4:1:1 nM ratio. All products were normalized to 0.2 ng/µL. 

Normalized lrPCR was utilized as a template to establish the primer-specific, circular long-range amplification sequencing workflow. Long-range PCR product from each of the 35-qPCR-positive samples was used as starting material in an Illumina Nextera XT library preparation that was performed following the Nextera XT Library Preparation Reference Guide (CT# 15031942 v01) using the Nextera XT Library Preparation Kit (Illumina, San Diego, CA, USA). Final libraries were analyzed for size and quality using the Agilent BioAnalyzer with the accompanying DNA 1000 Kit (Agilent, Santa Clara, CA, USA). Libraries were quantified using the Qubit HS Assay Kit (Invitrogen, Carlsbad, CA, USA) and normalized to 4 nM using 10 mM Tris, pH 8.5. Libraries were pooled and run on the Illumina MiSeq at a concentration of 10 pM with a 5% PhiX spike with run parameters of 1 × 150. 

### 2.4. Generation of Consensus Genome Sequences

MiSeq reads were imported into CLC Genomics Workbench (v. 8.5.1). Reads were trimmed of bases with greater than a 0.05 error probability and a maximum of three ambiguous nucleotides were allowed per read. Reads under 15 bases (10% of the average read length) after trimming were discarded. Reads were mapped to the previously described WSHBV genome (Accession# NC_027922) to generate consensus sequences for each sample. Read mapping required 75% of the length of the read to be at least 90% similar to the reference. A double-pass local realignment was also performed. The consensus sequence of the mapped and locally realigned sequences was extracted using quality scores, a noise threshold of 0.5, and a minimum nucleotide count of 2. Of the 35 samples, eight did not have >15x coverage across the complete genome, and these samples were not included in downstream analyses.

We then aligned 27 complete genome sequences (3541–3543 bp) produced by this resequencing method using a MUSCLE alignment in Geneious v. 9.0.1 (Biomatters, Auckland, New Zealand). Of the 27 sequences reported here, 10 had insertions or deletions (indels) in the alignment. To confirm the presence of indels, a consensus sequence from read mappings was generated using a noise threshold of 0.3 and a minimum nucleotide count of two. A MUSCLE alignment with eight iterations was performed between original consensus sequences and those with the lower noise threshold setting. 

Mapping and coverage analysis were performed in CLC Genomics Workbench and plotted using SigmaPlot v.13 (Systat Software Inc., San Jose, CA, USA). The 14 Sheboygan liver samples were not used in coverage analyses because they had been spiked with amplicons, thereby creating coverage biases. Circos was used to visually represent genome coverage patterns [16]. We also sequenced the previously published sample, RR173 (Accession# NC_027922), as a positive control to validate the MiSeq method, but did not use this resequenced genome in downstream analysis. 

### 2.5. Phylogenetics

The set of 27 sequences was tested for use with coalescent theory models using TempEst [17], which indicated insufficient temporal signal to proceed with BEAST molecular clock analysis. A larger sample set with a wider range of collection times for multiple subgenotypes would be needed to adequately calibrate evolutionary age estimations for these intra-genotype data [18]. When we included coho salmon kidney virus (CSKV), another parahepadnavirus [1], it was found to be dissimilar enough that resolution within the intra-genotype tree was lost. Intra-genotype variation evolves on a genealogical time-scale rather than a longer phylogenetic scale, suggesting that studying intraspecific data with inter-specific data could be misleading [18]. Bayesian phylogeny was, therefore, constructed in MrBayes v3.2.6 using three datasets of intra-genotype data from the WSHBV genomes sequenced here. We analyzed genome sequences, protein sequences, and a partitioned dataset containing genome nucleotide sequences and concatenated protein coding sequences. Model testing was performed in MEGA6 to identify the best nucleotide and protein models based on BIC score, which were HKY + G for genomes, JTT for the C and S ORFs, and JTT + G for the P ORF and concatenated protein sequences. Each of the three MrBayes analyses consisted of 1,000,000 MCMC generations, 6 chains, 4 independent runs, and 6 swaps. The chain temperature was set to 0.05 for genomes, 0.7 for individual proteins, and 0.07 for the partitioned dataset. The final trees were visualized in Dendroscope 3 (http://www-ab.informatik.uni-tuebingen.de/software/dendroscope, accessed on 11 January 2021) and edited in Adobe Illustrator CC. 

### 2.6. Variant Analysis

Populations were defined using sampling location in coordination with Bayesian trees, the measure of nucleotide diversity π (nucleotide diversity), and the genetic distance between populations, Dxy, calculated in DnaSP v5 [19]. Dxy was used instead of Fst, as the latter requires estimated haplotype frequencies, which could not be robustly determined from these sample sizes. GeneiousPro was used to identify single-nucleotide polymorphisms (SNPs) occurring between populations. Informative SNP sites were also confirmed in DnaSP v5. Variable sites by population were identified as transitions or transversions (ts or tv) and as synonymous or nonsynonymous substitutions (dN or dS) using GeneiousPro and DNAsp. The overall transition to transversion ratio, R (ts/tv) for each ORF was calculated using consensus sequences of each defined population under the Kimura 2 parameter model in MEGA7 [20]. In addition to defined population-specific SNPs, the high variance within the Athabasca River samples (π = 0.01864) versus the diversity within all Great Lakes samples (π = 0.00892) prompted us to record only nonsynonymous SNPs that were present in both Canada samples. Circos was used to generate circular figures [16] indicating SNP locations. 

### 2.7. Evolutionary Analyses

Phylogeographic haplotype consensus sequences were created from a MUSCLE alignment of relevant sequences for a total of five haplotypes. Evolutionary analyses were conducted using these consensus sequences and corresponding ORFs and conserved domains in PAML [21]. The modeled ratio of nonsynonymous to synonymous mutation rates (the parameter omega) was calculated for all sites of the individual protein coding ORFs, concatenated C-P-S ORFs, and spacer/PreS domains using codon substitution model 0 (M0). Sites under positive selection were then identified by comparing the likelihood of a codon substitution model that allows both purifying selection (omega < 1) and neutral evolution (omega = 1) with one that also allows positive selection (omega > 1). A shift in protein evolutionary rate between Great Lakes and Canada samples was examined by comparing the likelihood of a branch-specific model to the null model of equal rates along all branches. Statistical significance was determined by comparing the likelihood ratio to twice the value of the χ^2^ (chi-square) distribution with degrees of freedom equal to the difference in the number of model parameters, as recommended by the user manual. 

### 2.8. Haplotype PCR Assay

Primers amplifying the PreS/Spacer region unique to phylogeographic haplotypes were created using Geneious v. 9.0.1 (Biomatters, Auckland, New Zealand). Primers 613F 5′ GCACTCTTGGTTAGTTCATTCTGA 3′ and 1383R 5′ TCGAGGTTGGGGACTCTGTA 3′ were used to amplify a 771 bp product. Products were amplified using 1X GoTaq Green Master Mix (Promega, Madison, WI, USA), 0.4 μM of each primer, 1μL of DNA, and nuclease-free water to a volume of 25 μL. PCR amplification conditions consisted of 3 min at 95 °C, 30 cycles of 30 s at 95 °C, 30 s at 60 °C, and 1 min at 72 °C, and a final extension of 5 min at 72 °C. KAPA Pure Beads (Kapa Biosystems, Wilmington, MA, USA) were used in a 1.8X PCR clean up. Cleaned amplicon products were quantified on the Nanodrop (Thermo Fisher, Waltham, MA, USA), and normalized to 5 ng/μL using Tris-HCl, pH 7.5. Normalized amplicons were run on a 2% agarose gel (Sigma Aldrich, St. Louis, MO, USA) for 70 min at 90 V and imaged on an AlphaImager (Alpha Innotech, San Leandro, CA, USA). Cycle sequencing with 5 ng of normalized product was carried out using the BigDye Terminator v3.1 Cycle Sequencing Kit (Applied Biosystems, Foster City, CA, USA) according to manufacturer’s instructions. Sequencing was performed on the ABI 3130 (Applied Biosystems, Foster City, CA, USA). 

## 3. Results

### 3.1. Detection of WSHBV by qPCR and Comparison with Nanostring

The qPCR method we developed was effective for identifying WSHBV-positive fish, targeting a 115 bp region of the polymerase and surface coding region. The r^2^ of the combined standards for all four runs was 0.9925; each run individually had an r^2^ value ≥0.998. The efficiency of combined runs was 100.52%, and for individual runs it ranged from 99.9% to 100.7%. The dynamic range of the assay was between 10^0^ and 10^8^ copies per reaction with a conservative limit of detection of nine copies/rxn. The product melting temperature was 78.02 +/− 0.13. 

**Figure 2:**
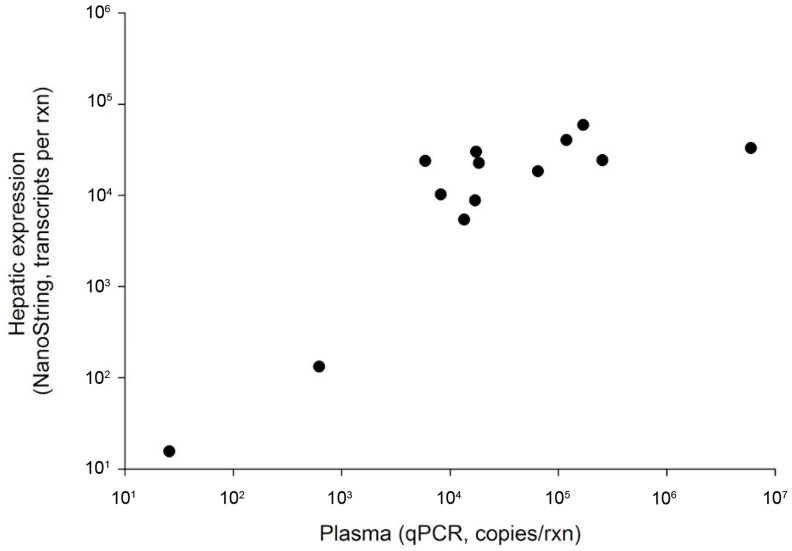
Comparison of detection of hepatic expression of WSHBV and viral presence in plasma. Hepatic expression was measured by Nanostring and plasma WSHBV DNA copy number by qPCR.

We identified 35 virus-positive fish. This included 19 of 219 fish using plasma only (8.7%) and 16 of 22 fish using liver only (72.7%). Virus-positive fish were identified at six of the seven Great Lakes Basin sampling sites. The WSHBV was not detected in fish collected from the Detroit River (Figure 1). In addition, 36% (4 of 11) of the white sucker plasma samples collected from the Athabasca River, Alberta, CA were WSHBV-positive. We had previously identified 13 of the 35 virus-positive fish via a Nanostring technology-based RNA-hybridization method [2]. Viral DNA was detected in plasma samples from all of these fish by qPCR (Figure 2). The Spearman correlation of log copy numbers by qPCR and Nanostring was significant (ρ = 0.7143; *p* = 0.0054). The absolute scale of copy number differed, as expected given that the qPCR utilized the DNA template from plasma while the Nanostring method targeted RNA in the liver. 

We initially sought to develop a minimally invasive, nonlethal approach that would facilitate the screening of wild captured fishes for robust epidemiological studies. We compared the detection of viral DNA in plasma and liver to determine if analysis of plasma alone was sufficient to identify virus-positive fish. We screened DNA extracted from the liver and plasma of 20 white suckers collected from the Sheboygan River during 2012. We identified 13 (65%) virus-positive individuals using template from the liver. Of these positive fish, only four (31%) were virus-positive based on screening plasma. Liver tissue was always positive for individuals in which viral DNA was detected in the plasma. While there were significant correlations in virus detection between plasma and liver samples (Pearson (ρ = 0.627, *p* < 0.003) and Spearman (*p* = 0.714, *p* < 0.001), viral DNA was not detected in the plasma of individuals in which liver copy number was less than 1.2 M copies/rxn. 

### 3.2. Long Range PCR and Amplicon Sequencing

Given the relatively small genome of the WSHBV, we developed a template-specific, long-range amplicon sequencing method to target 3319 bp of genomic sequence. Given the circular nature of these genomes, this targeted approach led to complete genome sequencing of 27 of the 35 virus-positive samples. We retrieved sequences of the targeted region for the other eight samples (23%; five plasma and three liver), but we defined these genomes as incomplete because coverage was less than 15X for >20% of the genome. Of the 27 that were fully sequenced, 18 plasma and non-amplicon spiked liver samples were used for coverage analysis. Average mapping coverage ranged from 16.7 to 133.5 K with a median of 53.2 K. The total reads per sample ranged from 520 to 3541 K with a median of total reads per sample at 1,510,536 for non-amplicon-spiked samples. The median percentage of total reads mapping to the reference viral genome for plasma samples was 96%. We observed a lower median percentage of reads (76%) that mapped to our reference genome when template DNA was from the liver. A higher percentage of mapped reads was observed for plasma or liver samples with the highest genome copy numbers of WSHBV, as determined by qPCR.

Coverage was not homogenous across genomes. The percentage of reads mapping to the atypical region (2536–3180) was 0.001 +/−0.0019% for plasma samples and 0.024 +/−0.0063% for liver samples, whereas the average percent of reads mapping to the rest of the genome was 0.0343 +/−0.0475% for plasma samples and 0.0292 +/−0.0135 for liver samples. The GC content in this region is also significantly lower, at 34% versus 42% of the rest of the genome. The reduced coverage may be a result of viral biology. Specifically, it may reflect the partially double-stranded DNA genome within virions versus the viral genome inside infected cells, which is predominantly cccDNA. Differences could also accrue from PCR artifacts such as chimeras and from GC-related biases during library preparation.

### 3.3. Phylogenetic Diversity 

**Figure 3:**
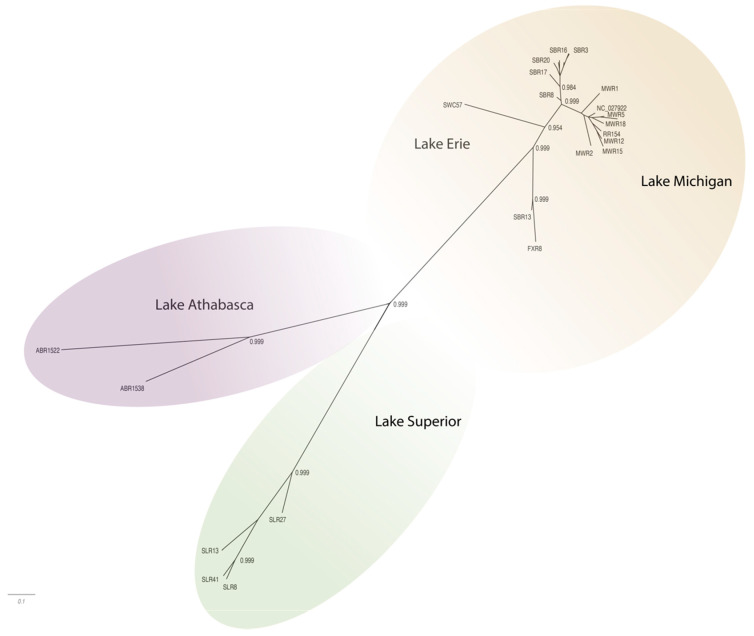
Radial phylogram depicting the relationships of 27 complete genomes (3541–3543 nt) of WSHBV isolated from white suckers inhabiting tributaries of the Great Lakes or the Athabasca River in Alberta Canada. Results are from Baysian analysis. Branches are labeled with posterior probability and color-coded by dominant clades. Isolates were tributaries of Athabasca Lake (Athabasca River, ABR) Lake Superior (St. Louis River, SLR); Lake Erie (Swan Creek, SWC) and Lake Michigan (Fox River, FXR; Milwaukee River, MWR; Sheboygan River, SBR and Root River, RR).

**Figure 4:**
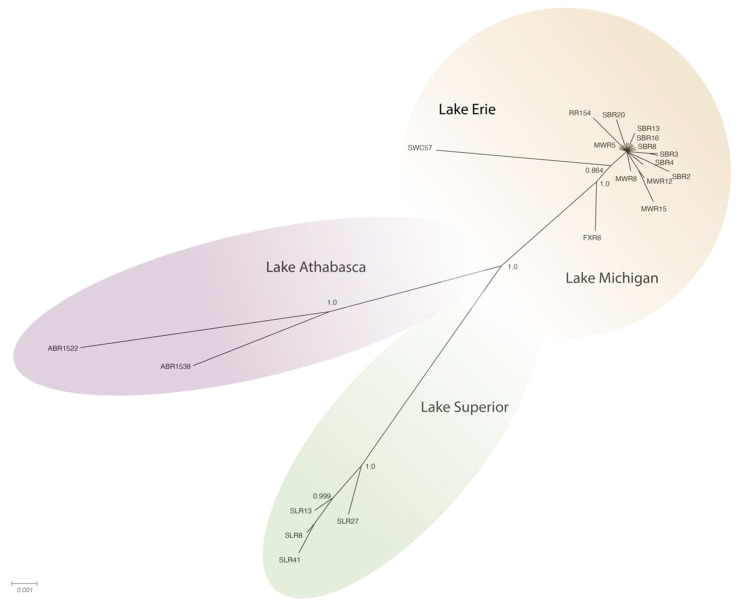
Radial phylogram depicting the relationships of concatenated protein sequences (P, C and S) from 27 complete genomes (3541–3543 nt) of WSHBV isolated from white suckers inhabiting tributaries of the Great Lakes or the Athabasca River in Alberta Canada. Results are from Baysian analysis. Branches are labeled with posterior probability and color-coded by dominant clades. Isolates were tributaries of Lake Athabasca (Athabasca River, ABR) Lake Superior (St. Louis River, SLR); Lake Erie (Swan Creek, SWC) and Lake Michigan (Fox River, FXR; Milwaukee River, MWR; Sheboygan River, SBR and Root River, RR).

The WSHBV genomes shared greater than 96.8% identity, and concatenated protein sequences shared greater than 96.9% identity. The greatest number of amino acid differences were observed in the P ORF (4.6%), followed by the S ORF (4.0%), and then the C ORF (1.9%; Table 1). The low divergence of the sequences is also evident in the topology of the phylogenetic trees, where branch lengths were generally short and only well supported in some cases (Figure 3 and Figure 4). The phylogenetic trees for individual genomes and weighted C, P, and S ORFs support similarly structured trees in which viral genomes from fish inhabiting the Athabasca River in Alberta, Canada were most distantly related to the group of Great Lakes samples.

Phylogenetic analysis of complete genome sequences identified isolates represented by three phylogeographically distinct clades. These included groups representing tributaries of Lake Athabasca (Athabasca River), Lake Superior (St. Louis River) and Lake Michigan (Milwaukee, Root, Fox and Sheboygan Rivers)/Lake Erie (Swan Creek; Figure 3; Supplemental Table S1). Within these phylogeographic groupings, the genome from Swan Creek (Lake Erie) and the single Fox River sample (Lake Michigan) formed a separate clade with a Sheboygan River sample based on complete genomic sequence (Figure 3). Phylogeographic groupings demonstrating evidence of geographic differentiation were better supported by the amino acid sequence of these three ORFs than nucleotide comparisons (Figure 4). Analysis of samples from tributaries of the Lake Michigan basin suggest inter-tributary transmission that represents a single composite region for subsequent haplotype characterization (Figure 3). 

While Canadian samples were phylogenetically distant from those of the Great Lakes samples, analysis of consensus protein-coding haplotypes using concatenated C, P and S ORFs (concatenated C-P-S) identified that the accrual rate of nonsynonymous substitutions is not significantly different across locations. However, the high similarity and small sample set limits the robustness of this test [22]. While WSHBV genomes representative of the Great Lakes and Canada samples had lower nucleotide divergence than the thresholds employed for orthohepadnaviral genotype demarcation, WSHBV variation was nonetheless geographically structured.

### 3.4. Haplotype Characterization

**Figure 5:**
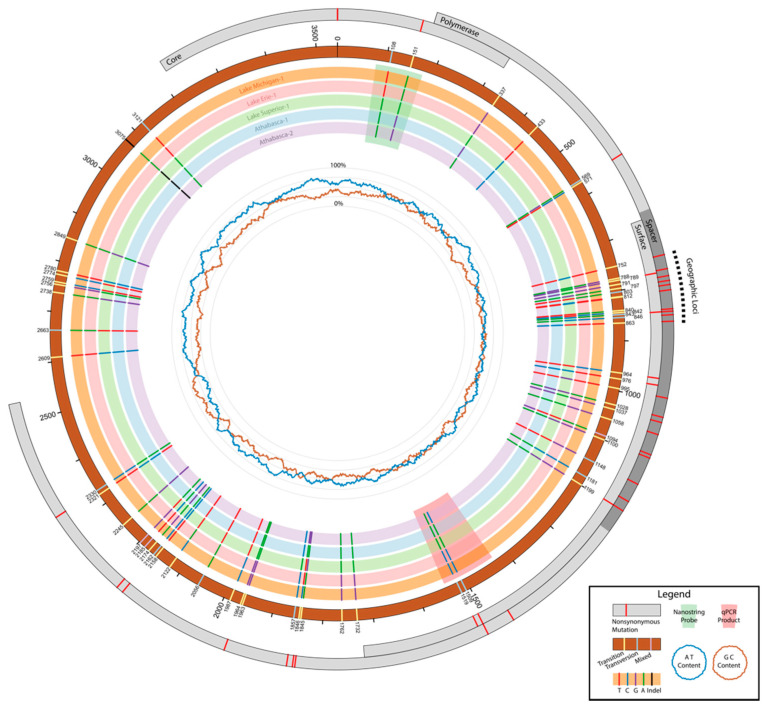
Polymorphic Sites across geographically structured haplotypes. The grey outer rings represent the protein-coding regions of white sucker hepatitis B virus (WSHBV) and are highlighted with nonsynonymous mutations. These mutations correspond with the single-nucleotide polymorphisms (SNPs) highlighted by the inner rings, which are representative of geographic sampling site. Small numbering around the positional reference ring also corresponds with these SNPs and shows whether changes are transitions or transversions. The inner most rings representing GC and AT content are on a scale of 0–100% (innermost to outermost).

Haplotypes were identified by examining nonsynonymous mutations relative to sampling location between all 27 complete genomes. A total of 215 polymorphic sites were identified, of which 131 were parsimony informative sites and 86 were sites of nonsynonymous mutations. Of the parsimony informative sites, 55 SNPs and two indels were identified as variable relative to sampling location. A total of 33 of 55 SNPs were missense mutations (60%). When the 10 indel sites identified by the workflow were re-examined, nine were recovered as real and occurred in eight sequences in three positions (2542, 3074, and 3184). Only two of the INDELs were geographically relevant. All three indel positions were within the atypical region, and therefore did not lead to frame shifts. Although SNPs were observed throughout the genome with some regions of apparent clustering, some of these regions of the genome were more prone to transversions and nonsynonymous mutations (Figure 5). We calculated the average nucleotide substitution per site (Dxy) values between sampling sites. The absolute pairwise divergence, Dxy, between sampling locations ranged from 0.00203 between the Sheboygan River and Milwaukee River to 0.03276 between Athabasca River-1522 and Swan Creek-57. The average Dxy was 0.022 (SD +/− 0.01). Consistent with the phylogenetic tree, WSHBV genomes from fish inhabiting the Lake Superior drainage were consistently more divergent from other Great Lakes samples. Canadian samples were even more different than genomes from the Great Lakes. The low divergence (Dxy < 0.007) between and within samples from the Milwaukee River, Fox River, Root River, and Sheboygan River supported collapsing sequences into a consensus for variant analyses as a Lake Michigan haplotype. Despite sequence similarity with the Lake Michigan samples, the genomes from Swan Creek (Lake Erie) was not collapsed to illustrate overall WSHBV haplotype variance. 

We further refined haplotype designation to the region spanning nucleotide 788–1199. This region contained SNPs that were used to define geographically informative haplotypes. Haplotypes defined here include Athabasca-1, Athabasca-2, Lake Superior-1, Lake Erie-1, and Lake Michigan-1. The genomic region with this phylogeographic signature was within the PreS domain of the S ORF and the spacer domain of the P ORF (Figure 5). This region is of evolutionary importance in other hepadnaviruses and has previously been used to classify genomic groups [23]. We calculated the percent nucleotide differences between C, P, and S ORF sequences and between the spacer in both reading frame (RF) + 2, corresponding to the P ORF, and in RF + 3 corresponding to the S ORF. Of the P ORF nonsynonymous mutations, 67% were within the spacer, and of the S ORF mutations, 70% were within the PreS. Comparatively, only 7.4% of P ORF mutations occurred within the RT-S overlap, whereas 30% of S mutations occurred in RT-S overlap. 

We developed a primer set to amplify and sequence this geographically informative locus for use in future studies to rapidly identify WSHBV haplotypes by conventional PCR. Sequences consistent with the amplicon sequencing results were recovered from samples from each haplotype, except Lake Erie SWC-57. The Sanger sequence of SWC-57 indicated nine nucleotide differences compared to the mapped consensus sequence, perhaps suggesting the presence of yet another haplotype. This included a nonsynonymous mutation within the phylogeographic signature. As observed in other hepadnaviruses, the S ORF genome region phylogeny was concordant with whole-genome- and protein-coding phylogeny. When determining genotypes, subgenotypes, and strains, this putative immunodominant region may deserve particular consideration. 

### 3.5. Protein Evolution Comparison with Related Viruses

Consensus sequences from haplotypes were used to evaluate the concordance of WSHBV annotations and features with other hepadnaviruses. The S ORF had the highest relative rate of nonsynonymous substitutions (*w* = 0.685), whereas the C ORF is most highly conserved (0.054), as observed in other hepadnaviruses [24,25,26]. The P ORF had an intermediate rate of amino-acid change (*w* = 0.286), within which the viral polymerase segment had an estimated ω = 0.092). 

As the spacer domain of the P ORF and the S ORF has been shown to accumulate nonsynonymous mutations under positive selection in hHBV [24,26], and exhibit codon-position biases, we tabulated the number of nonsynonymous substitutions by region and codon position. Within our sample of 27 WSHBV genome sequences, we identified 23 nonsynonymous nucleotide substitutions changing 25 amino acids within the pre-S/spacer overlap region (Figure 5). These were most likely to change the polymerase protein through the changing of codon position 1 of the P ORF and position 3 of the S ORF (p1/s3). The 21 p1/s3 mutations in the P ORF only accounted for two changes to the S ORF (Table 2). There were more via p1/s3 overall, but within the spacer p2/s1 and p3/s2 substitutions were similar in number/percentage. This differs from the pattern on other hepadnaviruses, in which p2/s1 mutations are less frequent (Table 2). Variation at the p2/s1 sites occur less frequently in other hepadnaviruses, probably because they change amino acids in both proteins [27]. Here, p2/s1 mutations in the P and S ORFs were not less likely than p3/s2 mutations but rate differences may become apparent as more WSHBV genomes become available.

### 3.6. Predicted Domains and Regulatory Elements

We predicted functional regions based on a combination of similarity with other hepadnaviruses and PROSITE Motifs. We also identified a single small ORF in the atypical region that overlaps the P ORF. Several functional nucleotide motifs were conserved among all WSHBV haplotypes. Two sets of 12bp direct repeats were identified within the atypical region, one set at positions 2519 and 3186 with the sequence 5′ CCATGTGCTCAC 3′, and the other at positions 2890 and 3126 with the sequence 5′ TGAAAGTCCATT 3′ (Supplemental Table S2). The function of these repeats is unknown in WSHBV. The noncanonical polyadenylation signal TATAAA, found in other hepadnaviruses and identified as the functional signal in WSHBV [2], was also conserved at position 3211 (Supplemental Table S2). 

Putative domains and enzymatic active sites were identified in the C, P, and S ORFs. Amino acids conserved across viral reverse transcriptase [1] were also conserved among haplotypes. This includes the glycine residue, the substitution of which is associated with orthohepadnaviral vaccine escape [28], and the YMDD motif of the reverse transcriptase domain that is conserved among other reverse-transcribing viruses [1]. Other conserved motifs of the reverse transcriptase (RT) were as follows: the active site, nucleic acid binding sites, and nucleoside triphosphate (NTP) binding site (Supplemental Table S2). In addition, Core Motif I and Core Motif II conserved in hepadnaviruses [3] were fully conserved throughout all haplotypes; Core Motif III was conserved in amino acids conserved across the family, but was variable at amino acid position 160 (nucleotide position 151) between the Great Lakes and genomes from the Athabasca River, Canada (Supplemental Table S2).

## 4. Discussion

WSHBV was the first hepadnavirus discovered in fish [2]. It was observed in liver of the freshwater teleost, white sucker (*Catostomus commersonii*). Since that initial discovery, other fish hepadnaviruses have been identified and classified [1,3,29]. Notably, the bluegill hepadnavirus was associated with a skin tumor. The WSHBV belongs to the genus *Parahepadnavirus*. This genus also includes the Coho Salmon Kidney Virus (CSKV). Additional hepadnaviruses that infect fish and amphibians, in addition to a novel group that lacks a lipid envelope, nackednavirus, have been discovered via in silico analyses of public short read archive databases. Deep-sequencing-based virus discovery projects are likely to identify pathogens that may explain idiopathic diseases or conditions that are otherwise inadequately explained. 

Here, we developed a qPCR method than can be utilized on plasma or liver samples to screen for the presence of WSHBV. While liver was the preferred tissue for evaluating prevalence, this method was effectively used to screen plasma from non-lethally sampled white sucker with high-titer WSHBV for the purpose of evaluating conditional prevalence and identifying samples for further genome sequencing or simple haplotype identification. The absence of viral DNA in plasma samples from liver-positive individuals is not unprecedented and likely depends on infection stage and disease progression [30,31,32,33,34,35,36]. While viral life cycle and host responses may, in part, explain our plasma detection limits, the screening of plasma for epidemiological studies may have value within the context of disease status once uncertainly limits are better established. Modifications to this method including increased sample volumes for plasma extraction, likely to improve assay sensitivity and utility in a non-lethal screening format. Such an approach would greatly increase sample size to better address phylogeographic variation in prevalence and haplotype. 

While the primer-specific lrPCR method utilized here was not originally designed for complete genome amplification, it enabled us to acquire full-length WSHBV genomes for 77% of the samples. The method provides a low-cost solution to complete viral genome re-sequencing and is amenable to multiplexed analysis [37]. There were limitations with the method in regard to comparatively lower coverage of the atypical region in plasma samples and between the primer-binding sites in liver samples. These may be the result of viral biology and/or sample type. Full-genome sequencing of haplotypes indicated that sample type rather than haplotype contributed more significantly to full genome sequencing ability. Given that the repertoire of genomic diversity is still uncharacterized, less sequence-specific rolling circle amplification methods may provide a more unbiased approach to full-genome enrichment.

Previous research of orthohepadnavirus genotypes has revealed adaptive mutations to occur most frequently within the overlapping P and S ORFs. More specifically, these changes are observed within the PreS domain of the S ORF and the corresponding spacer domain within the P ORF [1,27,38,39]. As the overlapping S and P ORFs have non-independent mutational constraints [24,40], it is unclear whether only one or both proteins are positively selected and with respect to what selective pressure. The PreS gene region is associated with host specialization of viral entry in both ortho- and avihepadnaviruses [7,24,41]. The recently described nackednaviruses lack this PreS/Spacer domain, perhaps allowing them to be generalists [1]. Human hepadnaviral Pre-S/Spacer domains were found to be under positive selection when several thousand isolates were fit to a customized two-frame substitution model [24]. 

Here we identified 57 variable sites within the genome. In particular, the 33 nonsynonymous mutations and indels serve as starting points to further study nucleotide site selection and functional protein changes in WSHBV. Biological properties of hepadnaviruses and those of the host likely contribute to viral genome variation, though neither factor alone is enough to explain patterns in genetic diversity [10]. Geographically differentiated viral strains are not unique to WSHBV. Human hepadnaviruses have a cosmopolitan distribution and are represented by 10 genotypes that are defined by phylogeographic nucleotide signatures [8]. Notably, these genotypes differ in prevalence, transmission, replication rate, serological profile, and clinical outcomes. The WSHBV genomes identified here do not satisfy the criteria defined for hHBVs to classify them as different genotypes. Despite this, there were observable nonsynonymous changes between five WSHBV haplotypes of Athabasca-1, Athabasca-2, Lake Superior-1, Lake Erie-1 and Lake Michigan-1. In addition, given the recently discovered diversity of hepadnaviruses, it is likely that conventional classification criteria need to be revisited and may differ among genera.

The observed protein coding changes between WSHBV haplotypes were similar to other hepadnaviruses in regard to genome position. Notably, the C ORF is the most highly conserved ORF within hepadnavirus species, and this appears to be the case for this parahepadnavirus as well. [42]. Interestingly, this ORF is highly diversified between hepadnaviral species. Under appropriate codon models, both the S and P ORFs of WSHBV had elevated nonsynonymous substitution rates that could reflect adaptive evolutionary pressures. The surface protein of hepadnaviruses is typically the most variable with the highest nucleotide substitution rate and ω values [25,38]. In particular, the spacer region of the P ORF and PreS in the S ORF are typically the most variable coding regions, and epitope shifts lead to immune system evasion with the highest number of substitutions in codon position 1 of the P ORF and 3 of the hHBV S ORF [24,26,38,43,44]. We observed these characteristics across WSHBV genome haplotypes, suggesting conserved functional strategies. Some of the haplotype SNPs may be explained by host and environmental factors. Fishes of different water bodies are exposed to a number of different stressors such as varying levels of contaminants [13,14,45] and/or different temperature, flow, and oxygen conditions. These varying environmental conditions can affect host genetics and immunity, and therefore the host viral response. White suckers live in pods, especially when young and during the day to avoid predators [46]. Each pod may have different inherent or adapted responses to general environmental conditions and/or to viral infection, which may affect viral selection and haplotype development. Haplotypes may alternatively be explained by variations in stages of the infection cycle, white sucker age at infection, and chronicity of infection [30]. Rather than a long rooted geographic signal in WSHBV, we may be observing SNPs selected from viral quasispecies by transmission bottlenecks, or those that offer viral advantages because of temporary colonization ability rather than adaptation.

White suckers migrate up to 40 river kilometers (rkm) into tributaries of the Great Lakes to spawn [47]. They return to preferred, restricted habitats within the Great Lakes where they reside for over 9 months of the year. This may provide an opportunity for pathogen mixing, thus leading to a phylogeographic signal with an underlying lake signature. Dilution of this signature may occur as the result of inter-lake transport of this fish for use as a baitfish, thus allowing introgression or support of multiple haplotypes. Based on the geographical expanse of this virus, and phylogeographical signatures, it is clear that this emerging pathogen has been established for considerable time. 

The haplotypes described here provide a foundation for future genetic analyses of WSHBV viral genome differentiation. Genotypes in human hepadnaviruses are determined using genetic diversity [48]; virulence and disease may vary within genotypes depending on site-specific mutations. Functional studies are required to address the significance of phylogeographically relevant mutations identified here. Once more is known about WSHBV biology, it will be conducive to study the relationship of haplotype infection with host responses to environmental exposure, the host basal immune system, the viral replication rate, and the host seroconversion rate. Understanding the host response to WSHBV is of particular importance in white sucker biomarker development and overall health assessments. The methods presented here offer a means to identify infected individuals and haplotypes to better understand WSHBV disease progression, epidemiological patterns and facilitate comparative hepadnaviral research.

## Figures and Tables

**Table 1:** Nucleotide diversity calculated by percent differences in pairwise identity.

	Core	Polymerase	Surface	Genome
Max % Nucleotide Differences	2.2	3.5	3.3	3.3
Max % Amino Acid Differences	1.9	4.6	4	
% Nucleotide diversity π (JC)	0.601	1.294	1.163	1.19

**Table 2:** Nonsynonymous substitutions by codon position of the overlapping Polymerase (P) and Surface (S) genes, restricted to the spacer region of P and pre-S region of S.

	p1/s3	p2/s1	p3/s2	Total Sites	Percent of Total nt Sites with dN Changes
Polymerase	21	6	0	27	1.45
Surface	2	3	5	10	0.96
Pre-S/Spacer	17	3	3	23	4.13
RT-S	1	1	2	4	0.83

## Data Availability

Genome sequences utilized for analyses are available in GenBank under accession numbers MW161131, MW161132, MW161133, MW161134, MW161135, MW161136, MW161137, MW161138, MW161139, MW161140, MW161141, MW161142, MW161143, MW161144, MW161145, MW161146, MW161147, MW161148, MW161149, MW161150, MW161151, MW161152, MW161153. MW161154. MW161155, and MW161156. The raw reads were deposited under BioProject PRJNA685065. Other data are hosted in ScienceBase at https://doi.org/10.5066/P9UF3YZJ, accessed on 11 January 2021.

## Supplementary Materials

The following are available online at https://www.mdpi.com/1999-4915/13/2/285/s1, Table S1: Metadata associated with complete WSHBV genomes, Table S2: WSHBV conserved functional domains and motifs.
