# Supplementary material for: Phylogeographic Genetic Diversity in the White Sucker Hepatitis B Virus across the Great Lakes Region and Alberta, Canada
Source: Viruses. 2021 Feb 12;13(2):285. doi: 10.3390/v13020285 (PMC7918172; doi:10.3390/v13020285)
Supplement: Supplementary file 1 [file viruses-13-00285-s001.zip › SuppleTable1.pdf]

Supplemental Table 1. Metadata associated with complete WSHBV genomes

Seqquence_ID	Isolate	Accession #	Country	State/Province	Haplotype	Collection Date	Sex	Length (mm)	Weight (g)	Location	Major Lake	Latitude	Longitude
CAN1538	ABR1538	MW161132	Canada	Alberta	Athabasca-1	9/18/12	M	461.00	1503	Athabasca River	Lake Athabasca	N 57.090407	W 111.556688
CAN1522	ABR1522	MW161131	Canada	Alberta	Athabasca-2	9/15/12	F	495.00	1686	Athabasca River	Lake Athabasca	N 57.029844	W 111.499812
GL2A_41	SLR41	MW161145	USA	MN	Lake Superior-1	5/23/11	M	447	894	St. Louis River	Lake Superior	N 46.736705	W 92.143833
GL16A_27	SLR27	MW161144	USA	MN	Lake Superior-1	5/2/12	F	488	1400	St. Louis River	Lake Superior	N 46.736705	W 92.143833
GL2B_13	SLR13	MW161146	USA	MN	Lake Superior-1	5/23/11	M	440	910	St. Louis River	Lake Superior	N 46.755409	W 92.116710
GL2B_8	SLR8	MW161147	USA	MN	Lake Superior-1	5/23/11	F	470	1204	St. Louis River	Lake Superior	N 46.755409	W 92.116710
GL3_57	SWC57	MW161148	USA	OH	Lake Erie-1	4/19/11	F	446	874	Swan Creek	Lake Erie	N 41.642611	W 83.552056
GL12_13	SBR13	MW161133	USA	WI	Lake Michigan-1	3/31/12	F	560	1562	Sheboygan River	Lake Michigan	N 43.753442	W 87.725283
GL12_16	SBR16	MW161134	USA	WI	Lake Michigan-1	3/31/12	M	493	1184	Sheboygan River	Lake Michigan	N 43.753442	W 87.725283
GL12_17	SBR17	MW161135	USA	WI	Lake Michigan-1	3/31/12	M	375	501	Sheboygan River	Lake Michigan	N 43.753442	W 87.725283
GL12_2	SBR2	MW161136	USA	WI	Lake Michigan-1	3/31/12	F	548	1995	Sheboygan River	Lake Michigan	N 43.753442	W 87.725283
GL12_20	SBR20	MW161137	USA	WI	Lake Michigan-1	3/31/12	M	422	727	Sheboygan River	Lake Michigan	N 43.753442	W 87.725283
GL12_3	SBR3	MW161138	USA	WI	Lake Michigan-1	3/31/12	M	460	1038	Sheboygan River	Lake Michigan	N 43.753442	W 87.725283
GL12_4	SBR4	MW161139	USA	WI	Lake Michigan-1	3/31/12	M	472	1067	Sheboygan River	Lake Michigan	N 43.753442	W 87.725283
GL12_6	SBR6	MW161140	USA	WI	Lake Michigan-1	3/31/12	M	467	1010	Sheboygan River	Lake Michigan	N 43.753442	W 87.725283
GL12_7	SBR7	MW161141	USA	WI	Lake Michigan-1	3/31/12	M	483	1088	Sheboygan River	Lake Michigan	N 43.753442	W 87.725283
GL12_8	SBR8	MW161142	USA	WI	Lake Michigan-1	3/31/12	F	411	881	Sheboygan River	Lake Michigan	N 43.753442	W 87.725283
GL12_9	SBR9	MW161143	USA	WI	Lake Michigan-1	3/31/12	M	493	1238	Sheboygan River	Lake Michigan	N 43.753442	W 87.725283
RR154	RR154	MW161156	USA	WI	Lake Michigan-1	4/23/14	F	525	1400	Root River	Lake Michigan	N 42.732451	W 87.811757
GL5_8	FXR8	MW161149	USA	WI	Lake Michigan-1	10/14/10	F	465	699	Fox River	Lake Michigan	N 44.573250	W 87.978972
GL6_1	MWR1	MW161150	USA	WI	Lake Michigan-1	4/12/11	M	458	1014	Milwaukee River	Lake Michigan	N 43.034440	W 87.910278
GL6_12	MWR12	MW161151	USA	WI	Lake Michigan-1	4/12/11	F	420	997	Milwaukee River	Lake Michigan	N 43.034440	W 87.910278
GL6_15	MWR15	MW161152	USA	WI	Lake Michigan-1	4/12/11	M	495	1252	Milwaukee River	Lake Michigan	N 43.034440	W 87.910278
GL6_18	MWR18	MW161153	USA	WI	Lake Michigan-1	4/12/11	F	440	899	Milwaukee River	Lake Michigan	N 43.034440	W 87.910278
GL6_5	MWR5	MW161154	USA	WI	Lake Michigan-1	4/12/11	F	466	946	Milwaukee River	Lake Michigan	N 43.034440	W 87.910278
GL6_8	MWR8	MW161155	USA	WI	Lake Michigan-1	4/12/11	M	387	589	Milwaukee River	Lake Michigan	N 43.034440	W 87.910278
