# Supplementary material for: Phylogeographic Genetic Diversity in the White Sucker Hepatitis B Virus across the Great Lakes Region and Alberta, Canada
Source: Viruses. 2021 Feb 12;13(2):285. doi: 10.3390/v13020285 (PMC7918172; doi:10.3390/v13020285)
Supplement: Supplementary file 1 [file viruses-13-00285-s001.zip › SuppleTable2.pdf]

Supplemental Table 2. WSHBV conserved functional domains and motifs

Nucleotide Range	Amino Acid Range	Motif/Domain/Sequence	Function
2370-2599	1-231 (RF + 3)	smORF3	unknown
2519, 3186	N/A	CCATGTGCTCAC	Direct Repeat - replication
2890, 3126	N/A	TGAAAGTCCATT	Direct Repeat - replication
3211	N/A	TATAAA	PolyAdenylation Signal
3159-3160	N/A	AG	Possible splice acceptor site
**Core Nucleotide Range (3216-315)**	**Core Amino Acid Range (RF +1)**		
3321-3359	36-48	LPHDFFPLLKDQV	Core Motif I
67-96	132-141	WWHTNCLMWG	Core Motif II
136-183	155-170	WLMTP(T/A)SYRNQYAPTI	Core Motif III
**Surface Nucleotide Range (684-1724)**	**Surface Amino Acid Range (RF +3)**		
684-1139	1-152	Pre-S*	
684-1166	1-161	Pre-S*	
1140-1724	153-346	S*	
1167-1724	162-346	S*	
**Polymerase Nucleotide Range (170-2536)**	**Polymerase Amino Acid Range (RF +2)**		
170-670	1-167	Terminal Protein (TP)	
332-340	55-57	GLY	Vaccine Evasion
671-1240	168-357	Spacer	
1241-2030	358-620	Reverse Transcriptase (RT)	
1430-1435, 1442-1444, 1571-1576, 1628-1639, 1769-1777	421-422, 425, 468-469, 487-490, 534-536	DL…A…FT…YMDD…FLG	RT putative active site
1430-1435, 1442-1444, 1571-1573, 1634-1636	421-422, 425, 468, 489	DL…A…F…D	Putative NTP binding site
1538-1540	457	G	Putative Nucleic acid binding site
1628-1639	487-490	YMDD	Protein priming and DNA synthesis
2030-2534	621-788	RH	
2087-2089, 2180-2183, 2249-2251, 2360-2363	640, 671, 694, 734	DEDD	Rnase H activity- coordinate metal ion binding (Nowotony et al., 2005)
2360-2372	731-734	NPAD	
